# Insulin Resistance Induced by Hyperinsulinemia Coincides with a Persistent Alteration at the Insulin Receptor Tyrosine Kinase Domain

**DOI:** 10.1371/journal.pone.0108693

**Published:** 2014-09-26

**Authors:** Karyn J. Catalano, Betty A. Maddux, Jaroslaw Szary, Jack F. Youngren, Ira D. Goldfine, Fred Schaufele

**Affiliations:** 1 Department of Obstetrics and Gynecology and the Diabetes Center, University of California San Francisco, San Francisco, California, United States of America; 2 Division of Endocrinology and Metabolism, University of California San Francisco, San Francisco, California, United States of America; Tohoku University, Japan

## Abstract

Insulin resistance, the diminished response of target tissues to insulin, is associated with the metabolic syndrome and a predisposition towards diabetes in a growing proportion of the worldwide population. Under insulin resistant states, the cellular response of the insulin signaling pathway is diminished and the body typically responds by increasing serum insulin concentrations to maintain insulin signaling. Some evidence indicates that the increased insulin concentration may itself further dampen insulin response. If so, insulin resistance would worsen as the level of circulating insulin increases during compensation, which could contribute to the transition of insulin resistance to more severe disease. Here, we investigated the consequences of excess insulin exposure to insulin receptor (IR) activity. Cells chronically exposed to insulin show a diminished the level of IR tyrosine and serine autophosphorylation below that observed after short-term insulin exposure. The diminished IR response did not originate with IR internalization since IR amounts at the cell membrane were similar after short- and long-term insulin incubation. Förster resonance energy transfer between fluorophores attached to the IR tyrosine kinase (TK) domain showed that a change in the TK domain occurred upon prolonged, but not short-term, insulin exposure. Even though the altered ‘insulin refractory’ IR TK FRET and IR autophosphorylation levels returned to baseline (non-stimulated) levels after wash-out of the original insulin stimulus, subsequent short-term exposure to insulin caused immediate re-establishment of the insulin-refractory levels. This suggests that some cell-based ‘memory’ of chronic hyperinsulinemic exposure acts directly at the IR. An improved understanding of that memory may help define interventions to reset the IR to full insulin responsiveness and impede the progression of insulin resistance to more severe disease states.

## Introduction

Insulin resistance, or the impaired ability of insulin to mediate glucose disposal, is a risk factor for several disorders including the metabolic syndrome, type 2 diabetes mellitus, gestational diabetes, cardiovascular disease and several forms of cancer [1]. Modifications in insulin signaling, often associated with imbalances in energy homeostasis such as obesity, have been linked to a predisposition towards the development of insulin resistance [2]. Once insulin resistance develops, the body responds through compensatory mechanisms designed to maintain insulin signaling. Here we examine how one of those compensatory alterations, an elevation in the concentration of circulating insulin, may itself cause a further decline in insulin signaling.

Insulin mediates its physiological effects by acting through a multimeric, transmembrane insulin receptor (IR) present at the surface of responsive cells. Once insulin is bound, the IR's intracellular tyrosine kinase domain becomes activated and phosphorylates specific tyrosines on the β-subunits of the IR dimer partners. This autophosphorylation initiates several signaling cascades that lead to insulin's downstream effects [3]–[7]. Insulin resistance could originate with a decreased amount of IR available to effect signaling. However, decreased overall IR levels are not typically observed in insulin-resistant patients with type 2 diabetes [8]. Furthermore, deficiencies in insulin signaling downstream of the IR have been heavily studied as a cause of insulin resistance in humans [9].

Insulin signaling also can be directly inhibited at the IR itself [2] as serine/threonine phosphorylation of the IR β-subunit, possibly through the protein kinase C pathway [10]–[13], inhibits IR tyrosine kinase activity [4], [14]. A direct inhibition of IR signaling also has been observed in mouse models in which insulin resistance is associated with a loss of IR phosphorylation upon elevation of protein tyrosine phosphatase 1B (PTP1B) [15]–[16]. Still further, an IR-interacting membrane glycoprotein, PC-1 (also called ENPP-1), has been implicated in insulin resistance and type 2 diabetes [17]–[21]; PC-1 seems to impair IR tyrosine kinase activity through a direct interaction of PC-1 with IR that does not affect insulin binding [22]–[23]. Thus, there is some evidence to suggest that some alterations at the IR itself may contribute to insulin resistance.

In individuals with functioning beta-cells, insulin resistance is often compensated for by increased beta-cell secretion of insulin. However, an elevated insulin concentration itself can induce or exacerbate insulin resistance [24]. For example, transgenic mice expressing multiple copies of the insulin gene, although lean and normoglycemic, exhibited marked insulin resistance [25]. Patients with primary insulinomas and no medical history of metabolic syndrome also have been observed to acquire insulin resistance, possibly as a result of their tumor-induced insulinemia [24]. Furthermore, diabetic patients receiving pulsatile, rather than continuous insulin infusion display better glucose control, suggesting that chronic insulin stimulation is best avoided for optimal insulin response [24]. While it might be tempting to suspect that an insulin-initiated turnover in IR could decrease the amount of IR available for signaling, most insulin resistant patients do not exhibit reduced skeletal muscle IR expression [26]. The mechanism by which an elevated or prolonged exposure to insulin (referred to here as hyperinsulinemia, regardless of origin) exerts its deleterious effects on insulin signaling remains unclear.

We examined whether prolonged insulin exposure induces a change in IR activity, membrane localization or structure that may render the IR less capable of being re-activated upon re-stimulation. Since the origins by which an individual may become hyperinsulinemic are variable, the studies presented here were conducted in cell culture models in which the amount and duration of insulin exposure could be carefully regulated independent of any other physiologic responses to hyperinsulinemia. The changes in IR structure and autophosphorylation detected in this study make it possible that the level and duration of insulin stimulation may act partially at the IR to initiate or progressively worsen insulin resistance.

## Materials and Methods

### Cell Culture

Rat hepatoma cells (HTCs) and Chinese Hamster Ovary (CHO) cells, obtained originally from the UCSF Cell Culture facility, were modified and maintained as described below. All cells were grown in Dulbecco's modified Eagle's medium containing 1 g/l glucose supplemented with 10% fetal bovine serum (Hyclone, Thermo Scientific, Waltham, MA, USA), 100 units/ml penicillin and streptomycin, and 1 mM glutamine at 37°C in 5% CO_2_.

HTC cells were genetically modified to express the cDNA for the B-isoform of the human IR (HTC-IR) [27], or the human IR fused at its C-terminus in frame with the yellow fluorescent protein (IR-YFP). The IR expression cassettes were co-integrated into the genome with a linked expression cassette conferring resistance to the antibiotic G418. The culture media for HTC-IR or HTC-IR-YFP stable cell lines was supplemented with 500 µg/ml G418 (Axenia Biologix, Dixon, CA, USA). The CHO cells were modified with either a PC-1 expression cassette linked to the G418-resistance expression cassette (CHO-PC1) or with the G418-resistance cassette only (CHO). Both CHO cell lines were maintained with 800 µg/ml G418.

### Insulin Treatments and Lysate Collection

For IR phosphorylation studies in HTC cells, extracts were prepared from cells grown in 10 cm plates to ∼80% confluence. On the day of the study, cells were washed twice with ‘starvation media’ containing no fetal calf serum or selection drugs but supplemented with 1% bovine serum albumin. Cells were serum starved for 4 hrs and then treated with recombinant human insulin (Sigma-Aldrich, St. Louis, MO, USA) at the indicated doses and times. At the conclusion of treatment, cells were rapidly washed with ice-cold PBS 3 times and solubilized in 0.5–1.0 ml of lysis buffer containing 50 mM HEPES (pH 7.6), 150 mM sodium chloride, 1% Triton X-100, 1 mM phenylmethylsulfonyl fluoride (PMSF), and 2 mM sodium orthovanadate at 4°C for 60 min. To remove cellular debris, lysates were spun at 14,000 g for 20 min at 4°C. Protein content was determined in each sample using the Pierce BCA protein assay kit (Thermo Scientific) per manufacturer's instructions.

### Determination of Total Insulin Receptor Tyrosine and Serine Phosphorylation

**Figure 1:**
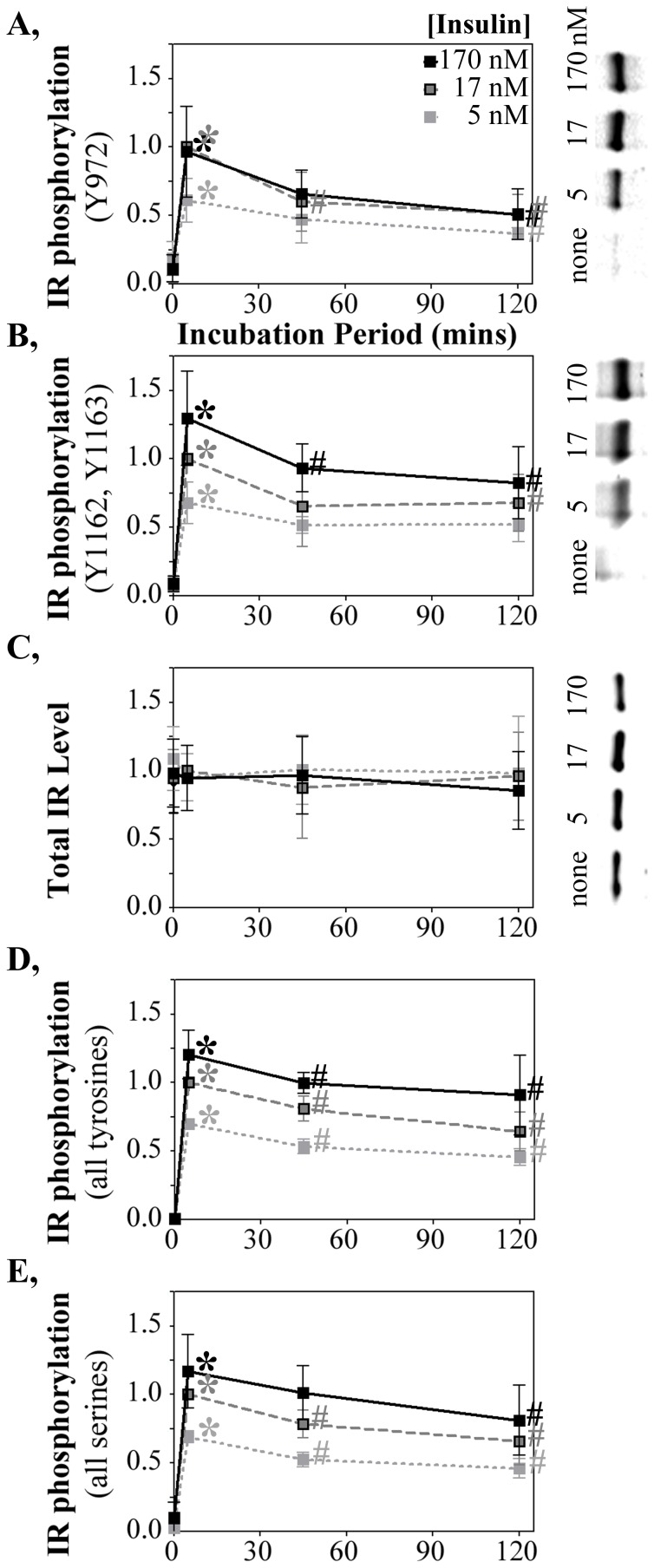
Chronic insulin exposure down-regulates IR activity. Timecourse and insulin dose response of IR phosphorylation at (**A**) pY IR^972^ and (**B**) pY IR^1162,1163^ detected by western blot with phospho-specific rabbit polyclonal antibodies. Detection and quantification was by infrared immunofluorescence linked to the anti-rabbit secondary antibody. Representative westerns shown for a dose response (right panels; only shown for the shortest insulin incubation period). (**C**) Total IR protein quantified on the same western blots with an anti-IR mouse monoclonal antibody conjugated with an anti-mouse secondary antibody linked to a distinct infrared fluorophore. Total (**D**) tyrosine and (**E**) serine phosphorylation, over all sites on the IR determined by ELISA, in response to insulin stimulation. Data are presented as mean ± sd from 3, 10 and 9 independent studies for the 5 nm, 17 nM and 170 nM doses. The intermediate 45 mins time point was included in a subset of those studies (3, 4 and 4 studies for the 5 nm, 17 nM and 170 nM doses). Since the absolute ‘light unit’ values quantified differ from experiment to experiment, the data were normalized to a treatment condition common amongst all experimental conditions. The earliest time point of induction (2–10′) with 17 nM insulin was set as 1.0 and all other data points within each study were normalized to that. *, increased IR phosphorylation (p<0.05) at the 2–10′ time point relative to the no insulin control. #, diminished IR phosphorylation at longer insulin incubation periods (p<0.05) relative to the 2–10′ time point. The data normalization and statistical symbols are applied similarly to the data presented in the remaining figures.

Total IR phosphorylation was determined using an enzyme-linked immunosorbent assay (ELISA) as previously described [28]. Equal amounts of cell lysates (20 µg protein) were loaded onto a 96-well plate coated with monoclonal anti-human IR antibody (MA-20, prepared in-house), that does not cross-react with the related receptor for insulin-like growth factor 1 [29]. Lysates were incubated with the IR-specific antibody for 18 hr at 4°C. Next, plates were washed with Tris-buffered saline containing 150 mM sodium chloride, 0.05% Tween-20, and 20 mM Tris (pH 7.4). Horse radish peroxidase (HRP)-conjugated mouse monoclonal anti-phosphotyrosine pY-20 (Santa Cruz Biotechnology, Santa Cruz, CA, USA) or rabbit polyclonal anti-phosphoserine (Abcam, Cambridge, England) antibodies were then added and allowed to incubate for 2 hr at 22°C; wells treated with the anti-phosphotyrosine antibody were subsequently incubated with horseradish peroxidase-linked streptavidin. Binding was measured upon the addition of the peroxidase substrate, 3,3′,5,5′-tetramethylbenzidine (TMB). The amount of tyrosine or serine phosphorylation was determined by reading the optical density (absorption at 451 nm) of the converted substrate. Measurements were conducted in triplicate and averaged. The means +/− standard deviation of the measurements from multiple independent studies are shown in the figures (see figure legend for numbers of studies). As absolute numbers vary from study to study, the data in the figures are shown normalized to a standard treatment condition common to all studies (see Fig. 1 legend).

### Western Blotting

Lysates (10 µg), prepared as described above, were diluted with an equal volume of 2× loading buffer and subjected to SDS-PAGE on a gradient gel (8–16%), followed by transfer onto a 0.45 µm nitrocellulose membrane. Membranes were blocked for 1–2 hr at room temperature in Odyssey Blocking Buffer (LI-COR Biosciences, Lincoln, NE, USA) and incubated overnight at 4°C with the following primary antibodies in blocking buffer containing 0.1% Tween-20: mouse monoclonal anti-IR (MA-20, 0.17 µg/ml final concentration), rabbit polyclonal anti-pY972 (Abcam, ab-5678, 1∶4000), rabbit polyclonal anti-pY1162,1163 (Santa Cruz, sc-25103, 1∶500), mouse monoclonal anti-ERK (Cell Signaling Technology, 1∶4000, Beverly, MA, USA), rabbit polyclonal anti-pERK (Cell Signaling Technology, 1∶2000), rabbit polyclonal anti-AKT (Cell Signaling Technology, 1∶2000), rabbit polyclonal anti-pAKT (Cell Signaling Technology, 1∶1000), rabbit polyclonal anti-GSK (Cell Signaling Technology, 1∶1000), and mouse monoclonal anti-pGSK (Cell Signaling Technology, 1∶1000). Membranes were then washed and incubated for 30 min at room temperature with 1∶10,000 dilutions of species-specific secondary antibodies (LI-COR Biosciences) conjugated with either 680 nm or 800 nm fluorophores. The secondary antibodies were removed by two washes of washing buffer and the fluorescence from each band was detected on the Odyssey Infrared Imaging System (LI-COR Biosciences). Fluorescence levels were quantified using Image Studio Software Version 2.1.10 (LI-COR Biosciences) with an in-lane background region used to subtract fluorescence values originating from noise. The means +/− standard deviation of those measurements from multiple independent studies (see figure legend for numbers of studies) are shown in the figures.

### Insulin Receptor Localization

Two days after seeding HTC-IR-YFP cells at 2,000 cells per well in 384-well tissue-culture-treated imaging plates (Greiner BioOne; Frickenhausen, Germany), wells were washed twice with starvation media and incubated in 30 µl starvation media for four hours prior to insulin addition. To each well was added 10 µl starvation media containing insulin diluted so that the final concentration was 17 nM insulin in 40 µl media. For control wells, 10 µl of starvation media without insulin was added. Images of the YFP-tagged IR were collected over a 6 minute period+/−3 minutes to the time points indicated in the figures. 30 minutes prior to image collection was added the membrane staining dye CellMask Orange and the nuclear staining dye Hoechst 33342 (Invitrogen, Carlsbad, CA, USA) to 2.5 µg/ml and 5.0 µg/ml final concentrations, respectively.

Images were captured with a 40×, 0.95 NA objective on an Olympus IX-70 microscope using a triple band-pass dichroic mirror and filter sets (Chroma Corp.; Brattleboro, VT) that distinguish, in order of image collection, the fluorescence of the YFP-tagged IR (excite 480–495 nm, collect 500–530 nm) from the CellMask Orange-stained membrane (excite 550–560 nm, collect 580–630 nm) and from the Hoechst 33342-stained nucleus (excite 365–395 nm, collect 435–465 nm). Image collection times were 1000, 25 and 50 ms, respectively. Filter wheels (Sutter Corp., Novato, CA, USA) rapidly move filters to collect sequential images within <200 ms of the end of the prior collection. Images were captured at 14-bit depth on a CCD camera (Andor Technology, Belfast, Northern Ireland) set at gain 1.5. Image collection was controlled by Metamorph 7.1 software (Molecular Device Corp., Downingtown, PA, USA).

Images were analyzed with Metamorph 7.1's multi-wavelength cell scoring module. Individual cells were identified by the nuclear marker, from which the cellular margins of each cell was established by the location of the membrane marker. The image analysis alogarithms best define membranes using a ‘negative’ of the CellMask Orange image (i.e., that image subtracted at each pixel from the maximal 14-bit values). Visual inspection shows that the cell margins defined by that alogarithm parallel a user's perception of the location of the stained membrane in the images although perhaps 50% of membrane segments show folds that do not precisely track with the alogarithm-defined cell margins. All membranes are included if the membrane margin is expanded 400 nm towards the interior of the cell. The IR-YFP intensities within that expanded membrane mask are shown in the figure. The IR-YFP intensities reported for the cytoplasm cover a cellular area that starts a further 600 nm interior to the membrane mask and do not include the cell nucleus defined by the nuclear marker.

### Förster Resonance Emission Transfer (FRET)

HTC or CHO cells were transiently transfected with a vector expressing a tandem repeat of cyan fluorescent protein (CFP) and YFP appended to the C-terminus of the IR (IR-CFP-YFP). Control transfections expressed either IR-CFP or IR-YFP. Cells first were plated into 6-well dishes containing sterile 22×22 mm Number 1 borosilicate coverglasses. Cells were grown an additional two days, then transfected using a total of 1.875 µg of each plasmid and 10 µl Lipofectamine LTC (Invitrogen) per well. Transfected cells were grown overnight following which cells were subjected to 4 hour insulin starvation prior to the initiation of insulin incubation (100 nM), as described in the prior sections. The cover slips were removed for imaging following insulin incubation for the times indicated in the figures. For some CHO studies, the remaining cells were collected for extract preparation and measurement of IR auto-phosphorylation by ELISA, as described above. Only in CHO cells was the transfection efficiency great enough to permit those autophosphorylation studies.

The ‘acceptor’ YFP (excite 490–510 nm, collect 520–550 nm), ‘donor’ CFP (excite 431–441 nm, collect 455–485 nm) and FRET (excite 431–441 nm, collect 520–550 nm) channels were collected on Hamamatsu (Tokyo, Japan) ORCA camera on an Olympus IX-70 microscope. Exposure times were 200, 400 and 100 ms, respectively, at camera gain 5. Excitation was with a 200 W Hg/Xe light source (OptiQuip; Highland Mills, NY, USA) that possesses a strong 436 nm peak optimal for exciting CFP.

FRET was calculated as previously described [30]–[31]. Briefly, when energy is transferred from the donor fluorophore (CFP) to the acceptor fluorophore (YFP), CFP intensities become dimmer whereas the amount of yellow light emitted upon CFP excitation (the FRET channel) increases. Because CFP and YFP both are independently excited by, and emit in, the FRET channel, the amounts of that ‘donor bleedthrough’ and ‘acceptor bleedthrough’ are determined from control cells transfected with only the CFP or YFP expression vectors. The background-subtracted and bleedthrough-corrected intensities in the donor and FRET channels are used to establish the percentage of donor energy transferred to the acceptor (E), on a microscope calibrated to define constants for that conversion. In the figures, E represents the mean +/− sd of multiple independent studies in which an average of 47 CHO cells or 27 HTC cells were collected for each measurement point in each independent study.

### Statistical Analysis

All Data were reported as mean ± sd from multiple independent studies. All studies were compared using a One-Way ANOVA with Tukey's post-hoc tests for individual comparisons. Statistical significance was set at p<0.05.

## Results

### Down-Regulation of Insulin Response Upon Chronic Insulin Exposure

To establish how the length of duration of insulin stimulation affects IR signaling, HTC-IR cells, in which the human insulin receptor is expressed and readily measured [22] were incubated with insulin at various doses and periods of time. Insulin responsiveness was measured first by the most proximal IR function activated by insulin following four hours growth of the cells in insulin-deprived media. Almost immediately upon insulin addition, activation of the IR tyrosine kinase results in tyrosine autophosphorylation of the IR at all insulin doses (5, 17 and 170 nM) examined (Fig. 1; *, p<0.01).

pY^972^ and pY^1162,1163^ are important sites for receptor activation that leads to further IR autophosphorylation and the binding/phosphorylation of IR interacting factors within the insulin signaling cascade. At all insulin doses, IR autophosphorylation at Y^972^ (Fig. 1A) and Y^1162,1163^ (Fig. 1B), measured by quantitative western blot with phospho-specific anti-IR antibodies, was induced within ten minutes of insulin stimulation and declined below that activated level at later time points (#, p<0.05). The changes in IR autophosphorylation were not accompanied by any changes in total IR amount (Fig. 1C). The progressive loss of IR autophosphorylation over time therefore was not a consequence of any diminution of the total IR pool.

The stimulation and down-regulation of IR global autophosphorylation also was evident by ELISA measurement of all anti-phospho-tyrosine antibody bound to insulin captured by an anti-insulin antibody (Fig. 1D). This suggests a bulk down-regulation in tyrosine phosphorylation scattered, across all 13 known IR autophosphorylation sites. ELISA studies of pan-serine phosphorylation of the IR showed a similar down-regulation (Fig. 1E). Thus, a general diminution of IR phosphorylation occurs upon chronic insulin stimulation.

There are similarities in tyrosine phosphorylation sites in the IR and the insulin-like growth factor-1 receptor (IGF1R). Some cross-reactivity has been reported for binding of the phosphotyrosine-specific IR antibodies used in figures 1A and 1B to both phospho-IR and phospho-IGF1R. The ELISA assay (Fig. 1D) is specific for the IR [29] and shows an insulin response pattern similar to that of the two phospho-IR/IGF1R-targeted antibodies. The apparent down-regulation of tyrosine phosphorylation upon chronic insulin exposure therefore seems to occur at multiple sites throughout the IR. With no change in IR level (Fig. 1C), the reduction in IR phosphorylation upon prolonged insulin exposure reflects an average loss of phosphorylation per IR, not a net loss in the amounts of IR present.

### Divergent Downstream Signaling Responses to Chronic Insulin Exposure

**Figure 2:**
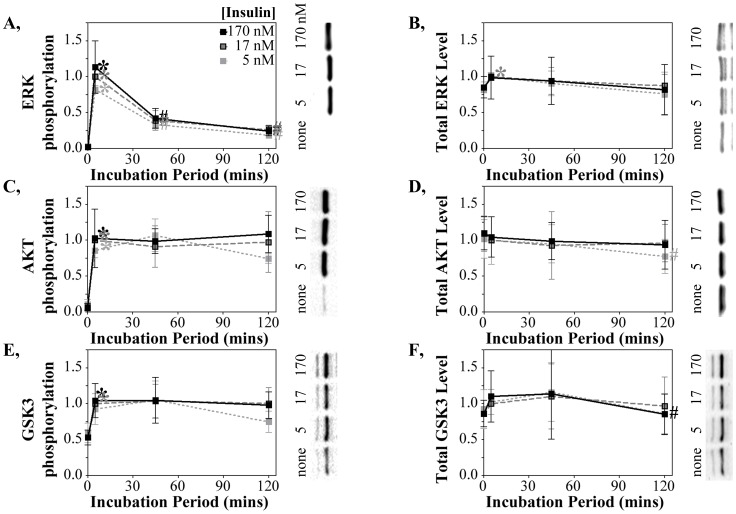
Differential down-regulation of downstream signaling pathways following chronic insulin stimulation. (**A**) phospho-ERK, (**B**) total ERK (**C**) phospho-AKT, (**D**) total AKT, (**E**) phospho-GSKβ, and (**F**) total GSKβ proteins quantified by western blot using the same extracts analyzed in figure 1. The fluorescence intensities of pERK and ERK, or pGSK and GSK, were quantified on the same blot with the phospho-specific and total protein antibodies (of rabbit and mouse origin) conjugated to specific-specific secondary antibodies with distinguishable fluorophores. The pAKT and total AKT antibodies both originated from rabbits and were probed in separate blots. Data normalization, number of studies, symbols and representative westerns are as described in figure 1.

The activation of IR autophosphorylation by insulin initiates a number of divergent signaling pathways, some of which were measured to define the consequences of phospho-IR down-regulation to downstream signaling. Phosphorylation of Erk1/2 at Thr202/Tyr204 was used as a read-out of insulin activation of the MAPK/ERK pathway. ERK showed a very strong and transient phosphorylation at 5 mins for all doses followed by a very rapid decline by 45 mins of insulin exposure (Fig. 2A). The changes in total ERK phosphorylation were not accompanied by any changes in total ERK protein (Fig. 2B). The activation of other insulin-regulated signaling pathways, measured by AKT phosphorylation at Ser473 and GSK3α/β phosphorylation at Ser21/Ser9, also reached maximum by 10 min at all insulin doses (Figs. 2C, E). Total AKT and total GSK3 protein levels similarly were unchanged by time or dose (Fig. 2D, F). No declines in AKT or GSK3 autophosphorylation were detected even 120 minutes after insulin addition (Fig. 2C, E). This does not necessarily mean that those signaling pathways are chronically activated as they may be down-regulated by other modifications at other sites.

### Insulin Refractory Response

**Figure 3:**
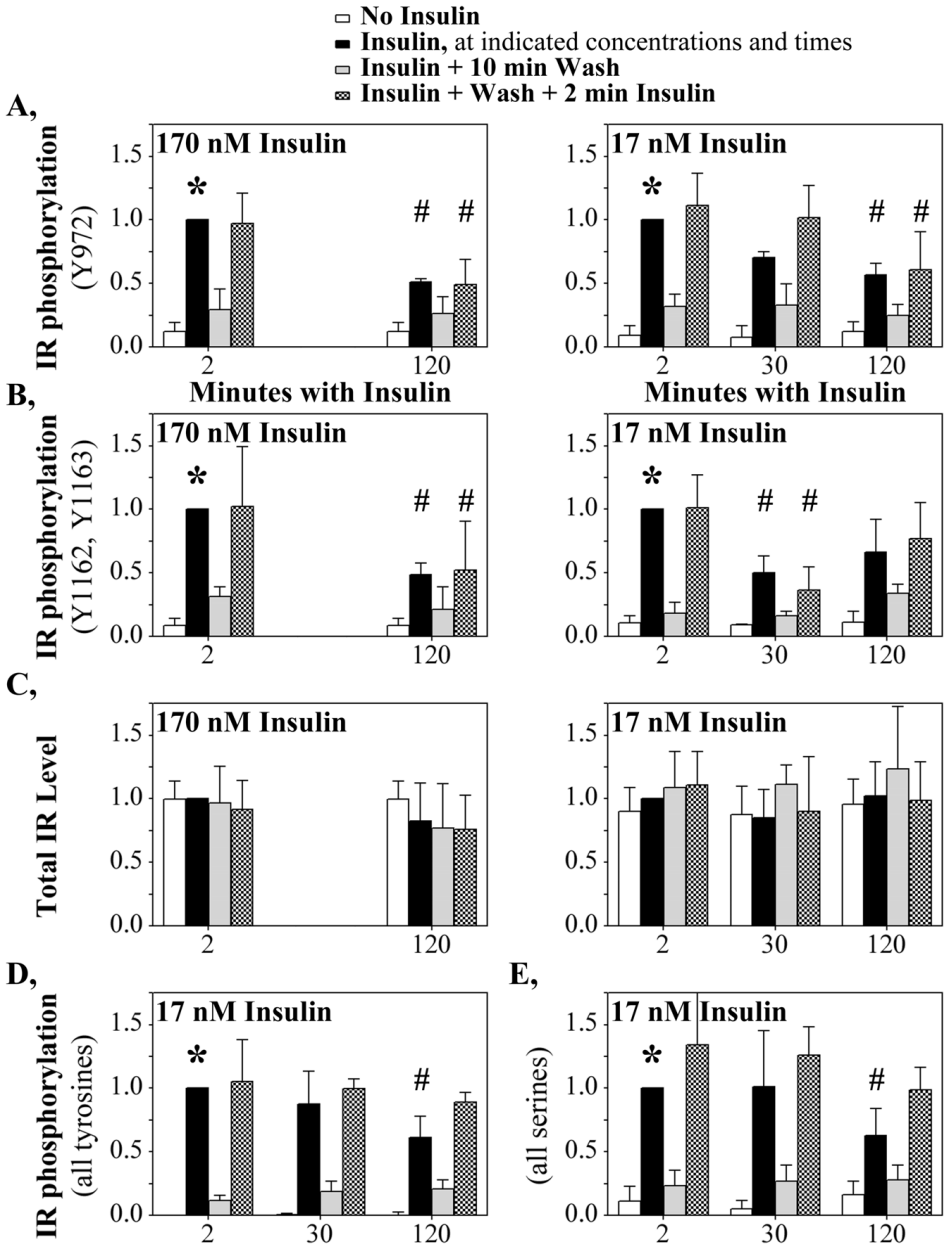
Chronically stimulated IR is refractory to subsequent re-stimulation following removal of insulin. Insulin receptor tyrosine phosphorylation at (**A**) pY IR^972^ (170 nM: n = 4 independent studies; 17 nM: n = 5) and (**B**) pY IR^1162,1163^ (170 nM: n = 4; 17 nM: n = 3) quantified by western blot in response to repeated insulin stimulation. (**C**) total IR protein quantified on the same blots. (**D**) Total tyrosine (n = 5) and (**E**) total serine (n = 5) phosphorylation, measured by ELISA (17 nM treatments). Data normalization and symbols are as described in figure 1.

The diminution of IR autophosphorylation following insulin stimulation may be a natural response that enables the IR to avoid an over-reaction to a lingering insulin stimulus. Once the remnants of that prior stimulation are erased, the expectation may be that the previously down-regulated IR will regain a full response to subsequent insulin stimulation. We therefore examined how chronic insulin stimulation affects the IR's ability to respond to subsequent insulin stimulation (Fig. 3).

HTC-IR cells were exposed for short (2 mins) or prolonged (120 mins) periods of 170 nM insulin (Figs. 3A–C, left panels) or 17 nM insulin (right panels), followed by removal of the insulin and a subsequent 2 min re-stimulation with the same insulin concentration. As in the earlier studies (Fig. 1), IR Y^972^ (Fig. 3A, black bars) and pY^1162,1163^ (Fig. 3B, black bars) phosphorylation was strongly elevated 2 minutes after insulin addition (*, p<0.05) and diminished by 120 minutes of insulin incubation (#, p<0.05). Following the primary 2 or 120 minute stimulation, the insulin was removed and the cells were incubated for only ten minutes with no insulin (gray bars). That washout of insulin was sufficient to erase most of the IR autophosphorylation. This rapid decrease in IR phosphorylation suggests that IR tyrosine phosphorylation is rapidly removed unless insulin remains present. Therefore, even at 120 minutes of chronic insulin stimulation, IR phosphorylation likely remains ongoing but is shifted to a lower level.

The critical question is whether, after wash-out of the insulin, the cell resets the IR so that the IR now responds to a second 2 minute stimulus (Fig. 3, checkered bars) equivalently to the initial 2 minute stimulus (black bars). Secondary insulin stimulation following 120 mins insulin exposure permitted the IR to regain only the diminished autophosphorylation level characteristic of the 120 mins time point. Thus, the conditions at the IR that caused it to have a diminished insulin response following 120 mins of chronic insulin exposure persisted after the wash. There was no change in the amount of IR present within the cell (Fig. 3C) that could account for that diminished response. By contrast, an initial exposure to only 2 mins of insulin was insufficient to diminish the second IR stimulation (Figs. 3A, B). This shows that the ‘insulin refractory state’ must first be achieved for the memory of that state to persist. As discussed later, even removing insulin for up to 180 minutes was insufficient to overcome the persistently diminished IR state.

For the 17 nM insulin studies, extracts also were collected 30 minutes following the initial insulin stimulation. Following 30 mins insulin exposure, IR phosphorylation at Y^1162,1163^ was already diminished and remained diminished upon insulin wash-out and re-stimulation (Fig. 3B). For Y^972^, the reduction in IR phosphorylation at 30 minutes of insulin exposure did not achieve statistical significance and the subsequent 2 mins insulin exposure was able to restimulate IR autophosphorylation to the level of the primary 2 mins stimulus.

Some differences in other phosphorylation responses were noted in the re-stimulation studies. Autophosphorylation across all IR tyrosines (Fig. 3D) or serines (Fig. 3E) was mostly or completely recovered by the ten minute wash-out of insulin. Thus, pY^972^ and pY^1162,1163^ are unusual amongst the insulin-induced phosphorylation sites in not rapidly recovering. Because pY^972^ and pY^1162,1163^ represent key sites in the initiation of the insulin signaling cascade, the refractory nature of re-stimulation at these sites may have an outsized effect on some aspects on a deadened insulin response when the IR is exposed to insulin for prolonged times.

**Figure 4:**
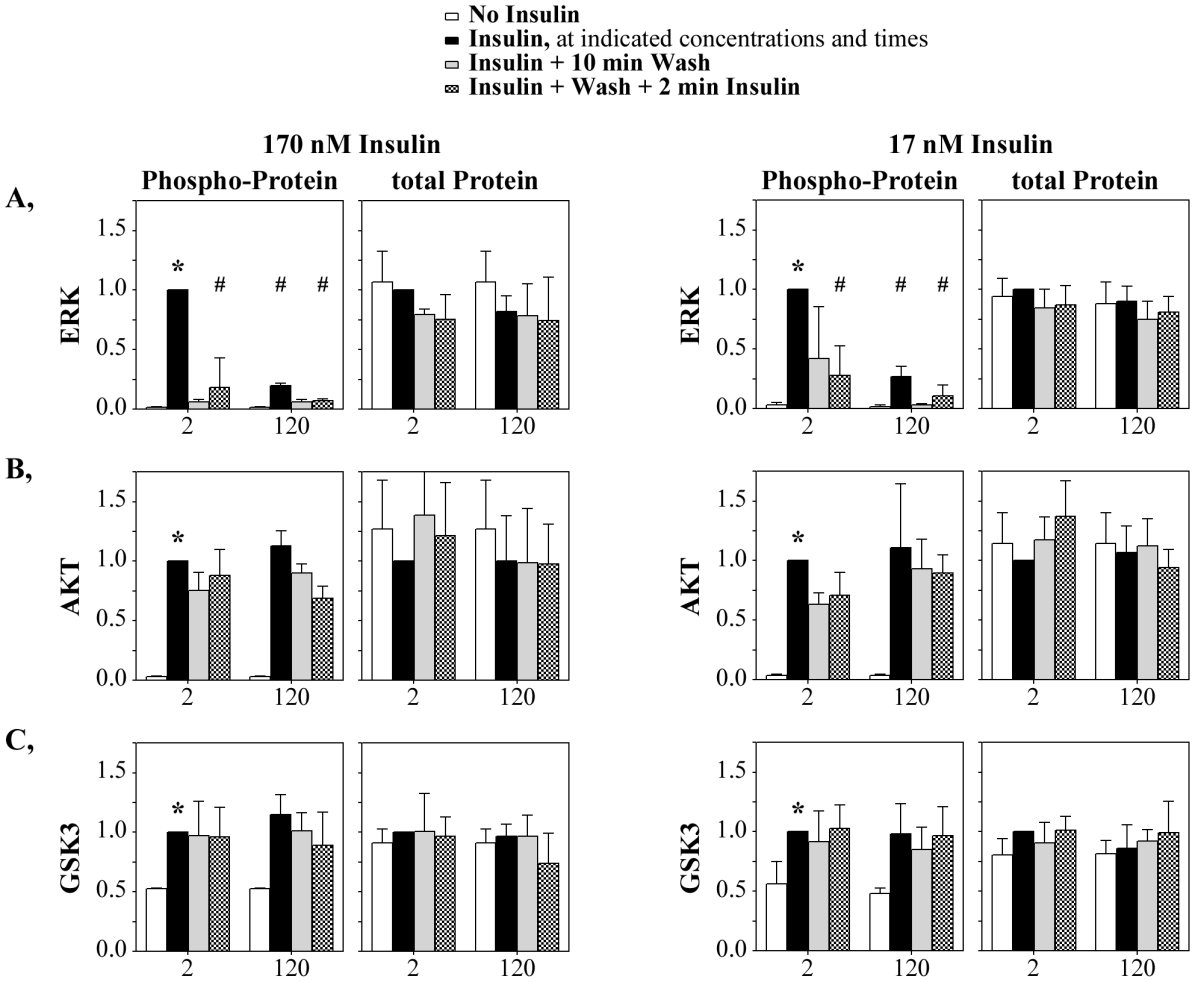
Differential impact of re-stimulation on pathways downstream of the IR. (**A**) pERK, (**B**) total ERK, (**C**) pAKT, (**D**) total AKT (**E**) pGSKβ and (**F**) total GSKβ proteins quantified by western blot in response to repeated insulin stimulation. See Fig. 2 for measurement details. Data are represented as mean ± SD from three (170 nM) and four (17 nM) independent studies. Data normalization and symbols are as described in figure 1.

The markers of downstream insulin signaling similarly were assessed for complete or partial recovery of the secondary insulin response following chronic insulin stimulation (Fig. 4). Erk phosphorylation could not be re-stimulated after a second 2 min stimulation following initial short- or long-term exposures (Figs. 4A, B; 2 mins or 120 mins). Thus, once stimulated by insulin, even for 2 minutes, Erk seems to be unable to respond to any secondary stimulation. Erk therefore appears to have rigidly controlled mechanisms of preventing secondary phosphorylation responses that are likely distinct from the more modest changes in IR pY^972^ and pY^1162,1163^ response to secondary stimulus following prolonged activation. Neither Akt nor Gsk3β phosphorylation was reduced by removing insulin (Figs. 4C–E) which is consistent either with sustained activity or a mechanism of down-regulation that does not involve removal of the activating phosphorylation.

### IR Internalization Does Not Account for the Refractory Response

**Figure 5:**
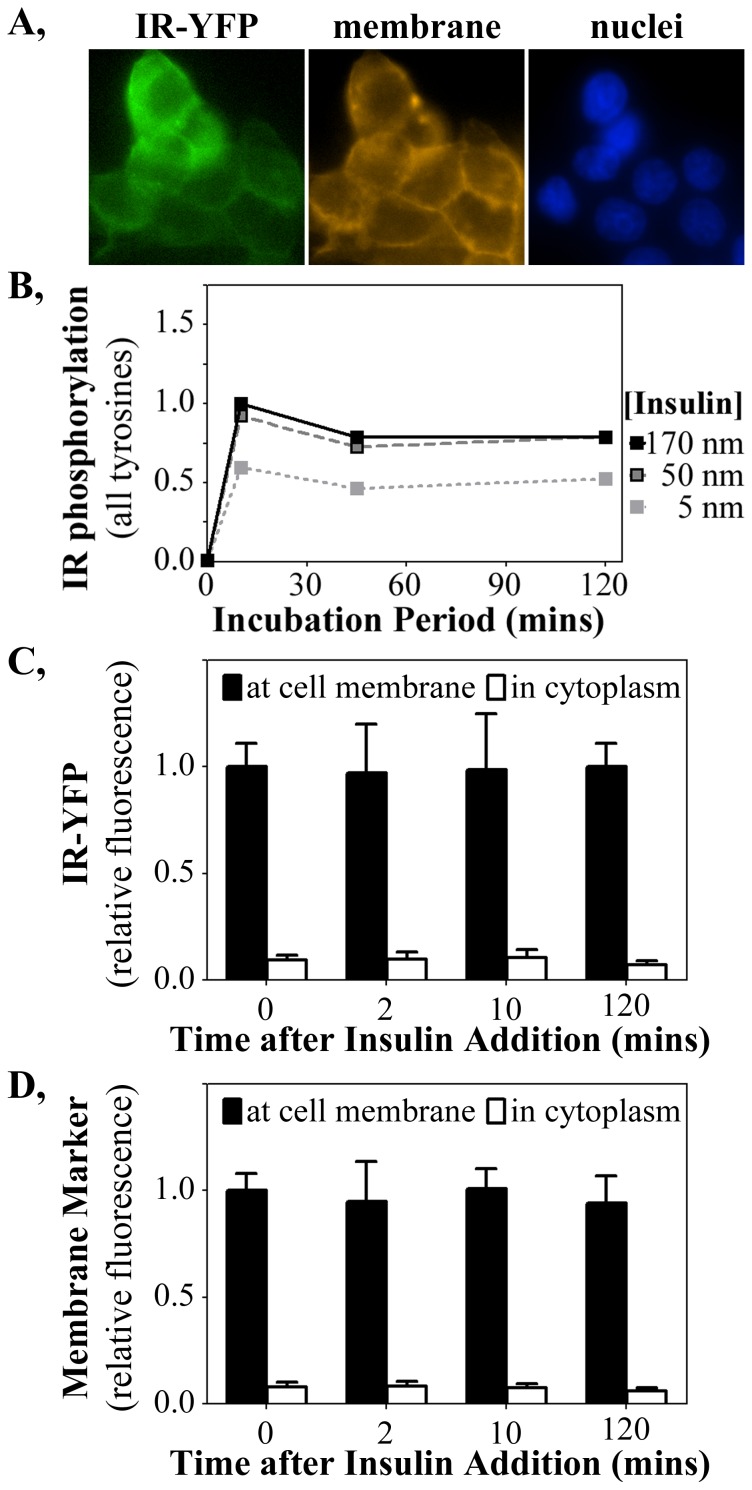
The IR remains membrane localized following chronic insulin stimulation of HTC-IR-YFP cells. (**A**) YFP fused to the IR fluoresces green and co-localizes with the membrane-specific stain (orange) and not with nuclei (blue). (**B**) Total IR tyrosine phosphorylation in HTC-IR-YFP cells, detected by ELISA, mirrors that of the unfused similar to HTC-IR in response to insulin stimulation (see Fig. 1D). The fluorescence intensity of (**C**) YFP (IR) and (**D**) the membrane marker were quantified at the membrane and in the cytosol of HTC-IR-YFP cells.

The studies of total insulin receptor level (Figs. 1C, 3C) showed no change in IR levels that could account for the diminished IR phosphorylation. However, long-term exposure to insulin could increase internalization of IR and sequester IR from the membrane to blunt insulin response. To test this possibility, HTC cells were created that stably expressed the cDNA for the human IR fused in frame at the C-terminus of the β-subunit with the cDNA for the yellow fluorescent protein (YFP). Fluorescence microscopy of these HTC-IR-YFP cells (Fig. 5A) showed that IR-YFP fluorescence coincided predominantly with cell membranes stained by the CellMask Orange membrane-specific dye. IR tyrosine autophosphorylation studies showed that the YFP-tagged IR responded to insulin dose and time-course studies (Fig. 5B) similarly to that measured for the untagged IR (see Fig. 1D). This HTC-IR-YFP cell line therefore was competent for comparing diminished IR autophosphorylation upon chronic insulin exposure with IR distribution in cellular membranes and cytoplasm.

Quantification of the amounts of IR-YFP at the cell membrane (marked by the membrane marker) and within the cytoplasm (Fig. 5C) showed that diminished IR phosphorylation induced by 17 nM insulin was not accompanied by any reduction in IR amount at the cell membrane. Similar measurements of the membrane marker (Fig. 5D) serve as a control for quantification accuracy and similarly did not change with insulin doses or incubation period. Less than 10% of the total YFP signal was detected in the cytosol, likely representing background, as quantification of the membrane stain showed a similar result. These findings make it unlikely that the strong reduction in IR phosphorylation after long-term insulin exposure arose exclusively from a reduction in IR availability to insulin at the cell membrane.

### The IR Tyrosine Kinase Domain Acquires an Altered State Upon Chronic Insulin Exposure

**Figure 6:**
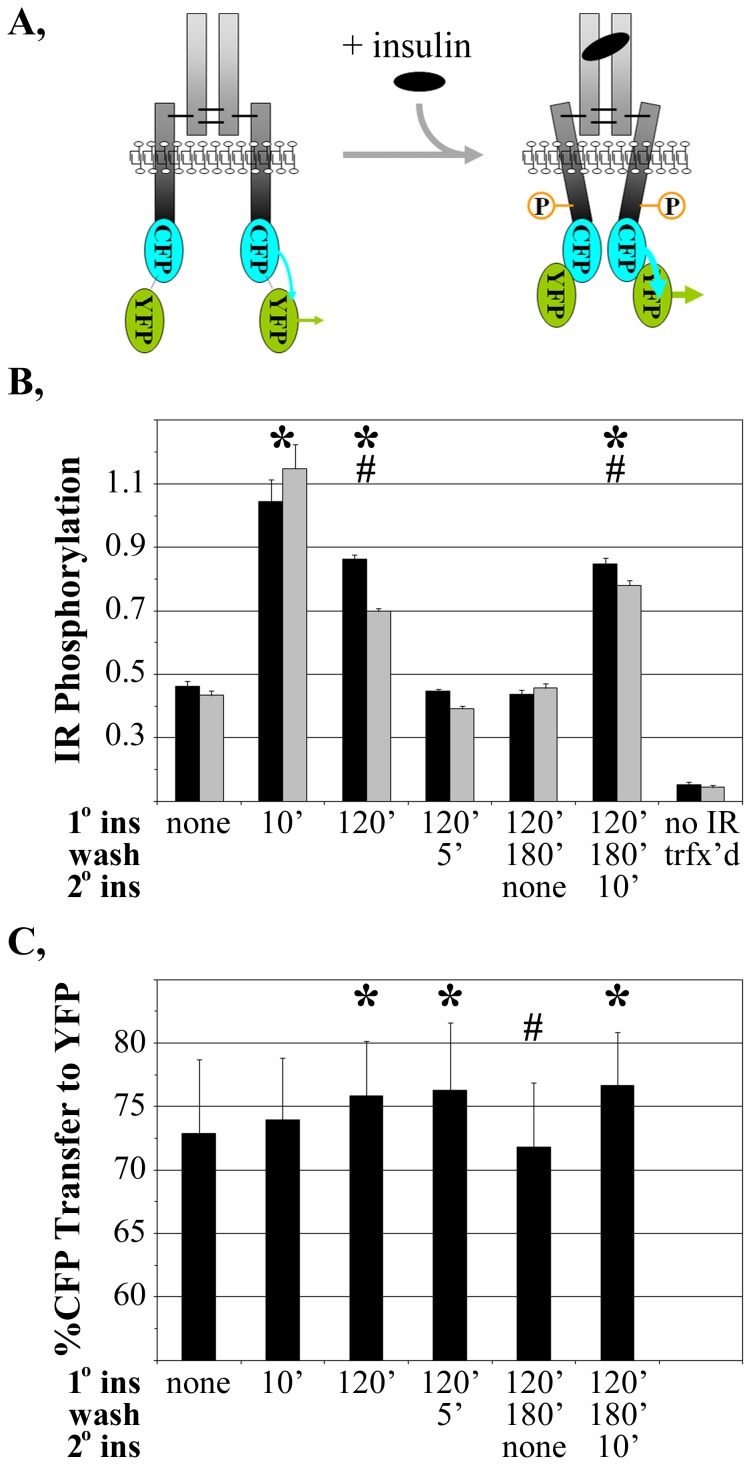
FRET measurement of altered IR structure or environment associated with insulin resistance in CHO cells. (**A**) Schematic representation of the IR-CFP-YFP probe and alterations in (**B**) total IR tyrosine phosphorylation or (**C**) energy transfer in that probe under acute or chronic insulin stimulation and after re-stimulation following insulin removal. The probe was transiently expressed in CHO cells. Symbols are as described in figure 1. Gray bars (Fig. 6B) represent IR phosphorylation levels normalized to IR expression levels determined by IR-CFP-YFP fluorescence.

The above studies showed that the insulin refractory response was not linked to changes in IR level or localization. We next investigated if it could be associated with any persistently altered tyrosine kinase (TK) state induced by prolonged insulin exposure. Changes in the structure of, or environment around, the IR TK domain were investigated by Förster resonance energy transfer (FRET). A human IR was tagged at the C-terminal TK domain with a fused CFP-YFP fluorophore and expressed in cells (Fig. 6A). Any insulin-induced conformational change, modification, or interaction with other factors, near the IR TK domain would shift CFP and YFP relative to each other. Such a shift would alter the percentage of CFP (donor) fluorophore energy transferred to the nearby YFP (acceptor) fluorophore.

Autophosphorylation studies were first conducted to assess if the IR-CFP-YFP probe remained functional. The autophosphorylation studies were conducted in CHO cells which were more readily transfected than HTC cells. Upon transient expression in CHO cells, IR-CFP-YFP was maximally phosphorylated within 10 min of insulin incubation (Fig. 6B, 10′); the level of autophosphorylation is presented per amount of extract (black bars) or adjusted for minor variations in transfection level (Fig. 6B, gray bars; normalized to YFP fluorescence from the IR). Both measurements showed that, as in the earlier studies in HTC cells stably expressing the untagged or YFP-tagged IR (Figs. 1, 3, 5), autophosphorylation of the transiently expressed IR-CFP-YFP probe became diminished after 120 mins of insulin exposure. Subsequent secondary insulin stimulation also permitted IR-CFP-YFP autophosphorylation only to the diminished ‘insulin-refractory’ level, even after washing away the insulin for 180 minutes. The IR-CFP-YFP probe thus retained the insulin refractory response characteristic of the non-tagged IR.

The availability of a functional IR-CFP-YFP probe allowed FRET investigation of alterations in the IR TK domain that might occur upon acute or chronic insulin exposure. The percentage of donor (CFP) energy transferred to acceptor (YFP) within the IR-CFP-YFP probe expressed in CHO cells was high (>70%) in the absence of insulin stimulation (Fig. 6C, none). This reflects the close proximity of the CFP and YFP attached in tandem to the most C-terminal amino acid within the IR. 10 minutes incubation with insulin did not alter that level of energy transfer (p>0.05). Thus, no gross change in the environment surrounding the IR TK domain was associated with the rapid induction of IR autophosphorylation.

By contrast, prolonged insulin stimulation increased (p<0.05) the amount of energy transfer (Fig. 6C, 120′). That suggested that the positions of the CFP and YFP tagged to the IR TK domain shifted upon prolonged insulin exposure. FRET changes may occur experimentally, as a consequence of the photobleaching of a fluorophore over repetitive excitation. That was avoided by measuring, for each time point, naïve cells grown on a separate slide and never exposed to light until a one-time, rapid automated image collection at the specific time-point. Thus, the data collection procedures minimized the potential for photobleaching artifacts. Furthermore, FRET increased at the 120′ insulin exposure time whereas photobleaching would have decreased FRET as a consequence of preferential photobleaching of the YFP ‘FRET acceptor’ that is more photolabile than the CFP ‘FRET donor’ [32].

A short wash-out of insulin was sufficient to permit the rapid loss of IR-CFP-YFP autophosphorylation (Fig. 6B, 5′ wash). However, that de-phosphorylated IR still maintained the ‘insulin refractory’ FRET level (Fig. 6C, 5′ wash) indicating that the altered fluorophore positions acquired upon chronic insulin stimulation was not linked to continued IR phosphorylation. It is possible that the persistence of insulin-refractory IR-CFP-YFP FRET may reflect an altered IR TK structure that constitutes a ‘memory’ state responsible for the reduced level of phosphorylation upon subsequent re-stimulation. However, that particular IR TK structure does not confer the memory state itself as the altered FRET state was expunged with a prolonged wash-out of insulin (Fig. 6C, 180′ wash) but still rapidly re-acquired with the subsequent 10′ re-stimulation.

**Figure 7:**
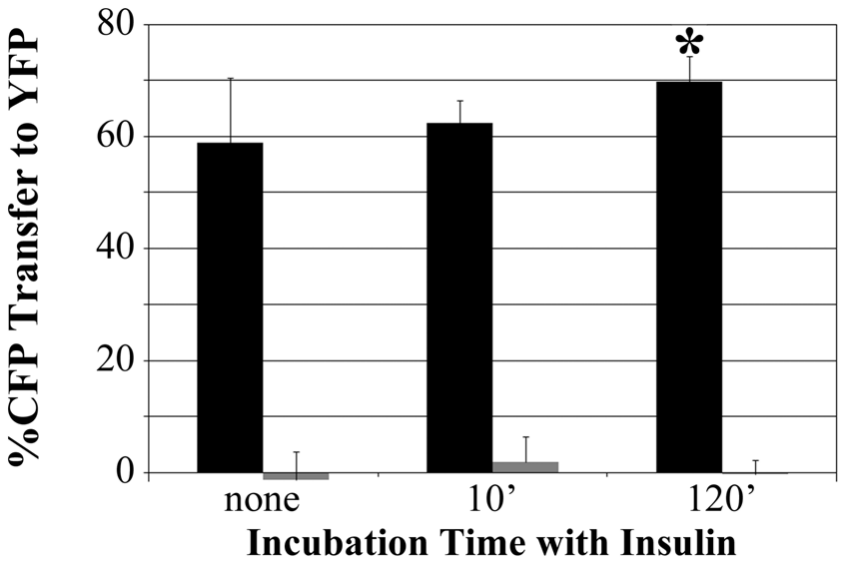
Insulin refractory IR structure in HTC cells. Energy transfer of IR-CFP-YFP probe transiently expressed in HTC cells subjected to acute (10′) and chronic (120′) insulin exposure. Gray bars, minimal energy transfer of CFP and YFP attached to the amino and carboxy termini of IRS1 (too far apart for energy transfer) demonstrates FRET measurement accuracy. Data represent the mean ± sd of the average FRET measurements of an average of 44 cells per point in each of three independent studies.

The insulin refractory FRET state also was observed when the IR-CFP-YFP probe was transiently expressed in HTC cells (Fig. 7, black bars). The baseline level of IR-CFP-YFP FRET in HTC cells was slightly lower than that in CHO cells. However, as in CHO cells, prolonged incubation of HTC cells with insulin increased the amount of energy transfer within IR-CFP-YFP (120′), whereas short insulin incubation did not (10′). As a negative FRET measurement control, the amounts of energy transfer also are shown for a probe in which CFP and YFP were attached to the amino- and carboxy-termini of the IR-interacting factor, IRS-1 (gray bars). That positioned the fluorescent proteins too far apart for efficient FRET which was accurately measured in the current studies.

### The Insulin-Refractory State Also is Acquired upon PC-1 Expression

Our prior studies also showed that insulin response, measured by IR autophosphorylation, was decreased upon the expression of PC-1 [33]. PC-1 is a membrane glycoprotein associated with insulin resistance in humans and in transgenic mice modified to over-express PC-1 [17], [19]–[21]. We therefore examined whether the shift in IR TK conformation, measured by IR-CFP-YFP FRET, occurred in the presence of PC-1.

**Figure 8:**
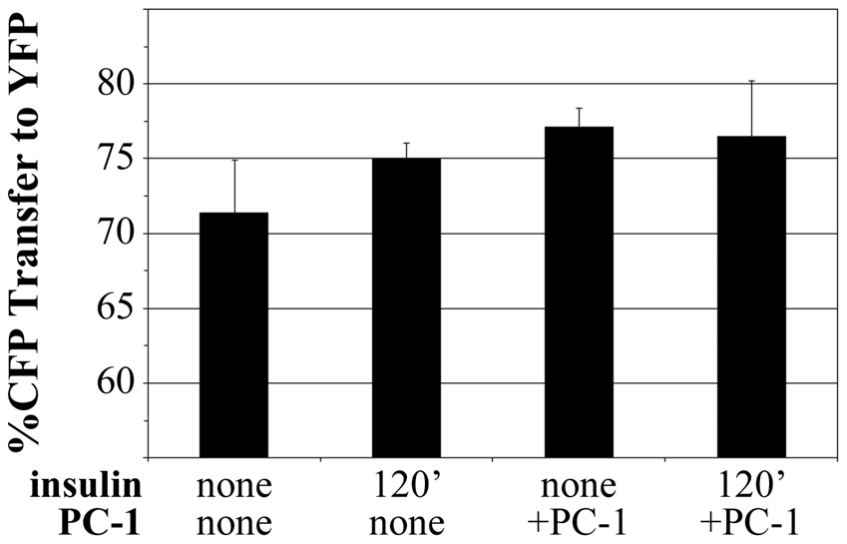
IR-CFP-YFP structure induced by chronic insulin exposure is similar to that induced by expression of PC-1. FRET levels of the IR-CFP-YFP probe transiently expressed in CHO cells or in CHO cells stably overexpressing PC-1. Measurements are conducted prior to insulin treatment or after chronic (120′) insulin exposure (n = 3 independent studies). Data represent the mean ± sd of the average FRET measurements of an average of 27 cells per point in each of three independent studies.

The IR-CFP-YFP probe was transiently expressed in CHO cell lines that stably expressed PC-1 linked to a G418-resistance cassette, or just the G418 cassette only (Fig. 8). Expression of the IR-CFP-YFP probe in PC-1-expressing cells resulted, even in the absence of chronic insulin exposure, in an elevated FRET signal reminiscent of that which occurs following chronic (120′) insulin exposure of cells not overexpressing PC-1. Statistical significance was not achieved in this study set, but the general trend is towards an elevated FRET level upon chronic insulin exposure (p = 0.068) or PC-1 overexpression (p = 0.066). The IR TK alteration detected by FRET thus may be a general marker of insulin resistance initiated by divergent mechanisms, including PC-1 expression or hyperinsulinemia.

## Discussion

That long-term or continuous insulin exposure could lead to insulin resistance is not a new concept. Insulin is normally released in low-amplitude pulses and rapidly cleared from the circulation [34]–[35] possibly so that the body avoids chronic insulin exposure. Indeed, a pulsatile delivery of exogenous insulin also appears to confer improved glucose control compared to continuous infusion of insulin [24], [36]–[41]. Given that insulin signaling must be exquisitely controlled to maintain metabolic control, it is logical that the body would possess mechanisms to prevent insulin signaling under conditions of insulin excess. Thus, in transgenic mice over-expressing insulin [25] or in patients with insulinomas [42]–[45], the resulting hyperinsulinemia is counteracted by a diminished insulin response. While such studies reveal a clear negative effect of hyperinsulinemia on insulin action, they do not elucidate the mechanism by which this process occurs so that it may be avoided.

We hypothesized that some deficit in IR activity may contribute to insulin resistance conferred by hyperinsulinemia. Once pre-incubated with insulin for prolonged times, the ability of IR to autophosphorylate in response to insulin became curtailed (Fig. 3). The deficit in IR phosphorylation following long-term insulin stimulation appeared to occur in the absence of any changes in either IR amount or membrane localization (Fig. 5). Notably, the refractory phosphorylation state occurred in concert with a distinct change, measured by FRET, in the position of FPs attached to the IR tyrosine kinase domain (Figs. 6, 7). The detection of the IR structural alteration in two cell lines and its parallel to the pronounced decrease in IR autophosphorylation upon prolonged insulin exposure suggests that the subtle FRET change measured may be affiliated with the down-regulation of IR signaling. The similar, subtle change in IR structure upon the induction of insulin resistance by another means (Fig. 8) further supports the structural/functional association although, as described below, this particular structure does not constitute the physical ‘memory’ that causes the retention of a diminished IR response characteristic of chronic insulin stimulation.

It has long been recognized that the kinetics of insulin binding to the IR are complex [46], possibly originating with alternative insulin binding sites within the IR [47]–[48] that may become more available with prolonged exposure. A lag time between insulin binding and an IR conformational change [49] similarly may constitute a component of a diminished IR autophosphorylation capacity upon prolonged insulin exposure. At the IR itself, insulin resistance has been associated with binding of PC-1 to the IR through a site in the IR [22] implicated in structural studies to act as a hinge that moves upon insulin binding [23], [50]. However, the relationship of the change in IR conformation measured here (Figs. 6, 7) to any of those events remains unknown. We also found that the altered insulin structure reported here and the lowered capacity for IR autophosphorylation were erased by a 180 min washout of insulin, but rapidly reformed thereafter. This suggests that the IR retained a memory of the insulin resistant state even after the phosphorylation mark and FRET structure were erased by long-term insulin removal. The physical origin of that memory thus remains unknown although it may be informative that the altered IR structure and impaired IR autophosphorylation may be similar to that observed upon overexpression of PC-1 (Fig. 8). Overall, the study suggested that persistent hyperinsulinemia induces a still poorly understood change in the IR or in factors binding to the IR that render it less efficacious following repeated stimulation.

Other studies showed that insulin resistance induced by inflammation is associated with insulin signaling events downstream of the IR. The development of insulin resistance is therefore expected to be complex and may occur at many levels. In the current studies of resistance induced by chronic insulin stimulation, we detected no dysregulation of the downstream effectors Akt or GSK3. Other studies at the IR itself suggest that insulin resistance may involve alterations in IR serine phosphorylation [4], [14] or in tyrosine phosphatase activities, possibly at Y^972^ [39]–[41], whose phosphorylation is critical for interaction with IRS-1 [51]. Indeed, in our studies of insulin resistance initiated by chronic insulin exposure, Y^972^ and Y^1162, 1163^ were refractory for re-stimulation by insulin (Fig. 3).

Overall, our data demonstrate an effect of long-term insulin stimulation that leads to a down-regulation of IR phosphorylation. That down-regulation appears to be associated with some change within the IR TK domain or in proteins interacting with the domain that persists after the insulin stimulus is removed. The prolonged wash-out studies in which the apparent insulin-refractory IR conformation was restored to its native state but was rapidly recovered upon re-stimulation, indicate that there is more to the mechanism than has been uncovered here. Our current hypothesis is that a molecular memory of prior chronic insulin exposure must consist of some lingering environment or transient interaction that remains primed, even after the insulin has been removed for 180 minutes, to rapidly affect IR conformation and phosphorylation. The persistence of this memory for so long has significant repercussions to the influence of our lifestyles and eating patterns on the development of insulin resistance. It also impacts discussions about optimal insulin delivery protocols for diabetic patients.
